# Analysis of Preventive Effect of Bisphosphonate for Osteoporotic Fracture in Patients with Alzheimer’s Disease and Patient Mortality

**DOI:** 10.3390/jcm14020300

**Published:** 2025-01-07

**Authors:** Min Uk Jang, Young-Min Kwon, Jihyun Hwang, Go Woon Choi, Min Seong Kim, Dong I. Lee, Sang Won Jo, Sung Jae Kim

**Affiliations:** 1 NV Brain Neurology Clinic, Seoul, Republic of Korea; mujang@gmail.com; 2 Department of Orthopedic Surgery, Hallym University Dongtan Sacred Heart Hospital, Hwaseong 18450, Republic of Korea; kwonym0814@gmail.com (Y.-M.K.); goni8586@gmail.com (G.W.C.); 3 Department of Biomedical Engineering, Johns Hopkins University School of Medicine, Baltimore, MD 21205, USA; jhwang50@jh.edu (J.H.); dlee120@jhmi.edu (D.I.L.); 4 Department of Integrative Bioscience and Biotechnology, Sejong University, Seoul 05006, Republic of Korea; minseongkim@sejong.ac.kr; 5 Department of Radiology, Dongtan Sacred Heart Hospital, Hallym University College of Medicine, Hwaseong18450, Republic of Korea

**Keywords:** osteoporosis, Alzheimer’s disease, fractures, bisphosphonates, long-term survival

## Abstract

**Background**: Alzheimer’s disease (AD) is the most common neurodegenerative disease in the older adult population and is often associated with reduced physical activity. Reduced activity and mechanical loading subsequently reduce bone mineral density and increase risk of osteoporosis. Bisphosphonates (BPs) offer preventative effects on osteoporotic fractures in the general population, but their effects on patients with AD are less known. This study aimed to assess the impact of BPs on osteoporotic fractures and survival in patients with AD. **Methods**: In this nationwide retrospective cohort study, 43,469 patients from the Korea National Health Insurance Service database between 2004 and 2018 were included. All patients were diagnosed with AD and subsequently diagnosed with osteoporosis. Continuous use of BPs was defined as having prescriptions for BP medications one year after the diagnosis of osteoporosis. Propensity score matching paired 12,519 BP users with 12,518 non-users for post-fracture survival analysis. **Results**: Continuous use of BPs showed a significant preventative effect on the Cox regression model [hazard ratio (HR), 0.890–0.895; *p* < 0.001] but not on the logistic regression model. The occurrence of osteoporotic fractures in the hip or spine significantly increased the risk of death [hip, HR, 2.036; 95% confidence interval (CI), 1.789–2.316; *p* < 0.001; spine, HR, 1.465; CI, 1.305–1.644; *p* < 0.001]. **Conclusions**: Continuous use of BPs was associated with reduced occurrence of osteoporotic fractures in patients with AD. Patients with AD showed significantly higher mortality rates after the occurrence of osteoporotic fractures. Further studies with detailed patient characteristics and compliance are warranted.

## 1. Introduction

Alzheimer’s disease (AD) is one of the most prevalent neurodegenerative disorders, mostly affecting the older adult population, characterized by progressive deterioration in cognitive functions and daily living abilities. AD severely impacts patients’ quality of life and overall survival [1]. In addition to cognitive decline, AD is often associated with reduced physical performance and activity, which increases the risk of falls and fractures [2]. These issues are particularly concerning as fractures can further contribute to morbidity and mortality in this vulnerable population [3].

Osteoporosis, a condition characterized by reduced bone strength and increased fracture risk, is closely related to physical inactivity in patients with AD [4]. Reduced mechanical loading on bones leads to reduced bone mineral density (BMD) and increases the risk [5,6].

Bisphosphonates (BPs) are a class of medications that inhibit osteoclast activity, reducing bone resorption and increasing BMD [7]. These drugs are widely utilized in the management of osteoporosis and have demonstrated efficacy in preventing osteoporotic fractures, particularly in the spine and hip [8]. Studies in the general population have consistently highlighted the fracture-preventive effects of bisphosphonates, but their efficacy in the specific context of AD remains underexplored [9,10].

The current literature on the use of BPs in AD suggests potential benefits in increasing BMD and reducing fracture risk [11]. However, data specific to this population still remain limited in the literature [12]. As AD patients represent a specific group with distinct pathophysiological characteristics and risk factors for fractures, further investigation is warranted to clarify the effectiveness of BPs in this context [13]. As a large-scale study conducted on a specific patient population vulnerable to osteoporotic fractures, the current study is expected to provide more scientific evidence to enhance the management.

This study aims to evaluate the impact of BPs on the prevention of osteoporotic fractures in AD patients and to analyze survival outcomes following osteoporotic fractures using Kaplan–Meier survival analysis based on fracture location.

## 2. Materials and Methods

### 2.1. Study Population and Design

This study utilized data from the Korea National Health Insurance Service (KNHIS). This comprehensive claim database includes diagnostic codes; prescription records; health examination data; and demographic details such as age, sex, residential area, socioeconomic status, and date of death. The KNHIS database covers approximately 98% of the Korean population, ensuring robust and representative data. Ethical approval for this study was obtained from the IRB/EC of Dongtan Sacred Heart Hospital (HDT 2020-05-001). All data were totally anonymized, and informed consent was waived.

The dataset provided by KNHIS included information on 2,392,585 individuals diagnosed with AD between 2004 and 2018. After applying inclusion and exclusion criteria, 43,469 patients were selected for the final analysis. The inclusion criteria for the study were patients diagnosed with AD and subsequently diagnosed with osteoporosis.

The exclusion criteria were patients diagnosed with AD before 2006, diagnosed with osteoporosis before their AD diagnosis, with a history of fractures before their osteoporosis diagnosis, without health examination data, and with missing data.

AD was identified using diagnostic codes, and osteoporosis was defined as having both a diagnosis of osteoporosis and a prescription for BPs. The date of the first osteoporosis diagnosis was set as the index date. Osteoporotic fractures were defined as acute fractures that occurred after the index date of osteoporosis diagnosis.

Among the 43,469 selected patients, 12,518 continuously used BPs, while 30,951 did not. Propensity score (PS) matching resulted in 12,518 BP users and 12,518 non-users for survival analysis. PS matching was based on variables such as sex, age, residential area, health examination results, and comorbidities.

Of the 10,306 patients who experienced fractures, 8710 with complete health examination data were included in the analysis of the impact of osteoporotic fractures on mortality. Survival analyses were conducted to evaluate the time from fracture occurrence to death, focusing on fractures in three specific regions: the hip, spine, and wrist.

### 2.2. Assessment of Continuous Exposure to BPs

BPs included medications in the etidronate, alendronate, risedronate, and ibandronate classes. Information on BP usage was derived from prescription records within the claims database. Due to data access restrictions, detailed information about specific BP medications was unavailable, and only the presence of BP prescriptions could be confirmed. From a clinical perspective, all osteoporosis patients were assumed to have been prescribed BPs for diagnosis. Continuous BP use was defined as having prescriptions for BP medications within ±90 days of the anniversary of the osteoporosis diagnosis.

### 2.3. Covariate Assessment

Covariates included basic demographic information, comorbidities, and health examination variables. Demographics include age, sex, residential area (urban vs. non-urban), and socioeconomic status. Socioeconomic status was classified into four levels based on insurance premium levels divided into 20 categories by KNHIS. Comorbidities with Charlson comorbidity index (CCI) conditions were included, along with additional comorbidities such as hyperparathyroidism, thyrotoxicosis, inflammatory bowel disease, ankylosing spondylitis, systemic lupus erythematosus, and multiple myeloma. Health examination data included height, weight, waist circumference, systolic and diastolic blood pressure, proteinuria, hemoglobin, fasting glucose, total cholesterol, HDL, LDL, triglycerides, AST, ALT, γ-GTP, smoking, alcohol intake, physical activity score (PAS), and low BMI. Smoking was categorized as non-smoker, ex-smoker, or current smoker. Alcohol intake was classified as heavy drinkers (>30 g/day for males, >25 g/day for females) or non-heavy drinkers.

Physical activity was assessed using the PAS, calculated based on the frequency of light, moderate, and vigorous exercise per week from health examination surveys. Each activity level was assigned metabolic equivalent task (MET) values of 3, 5, and 8, respectively. The PAS was calculated as follows:

PAS = (3a + 5b + 8c)/7,(1)

where a, b, and c represent the number of days per week of light, moderate, and vigorous activity, respectively. BMI was categorized as low if <18.5.

## 3. Results

The demographics of study subjects are summarized in Supplementary Table S1. The total number of subjects included in current research in the unmatched sample set was 43,469; after PS matching, the total number of subjects included was 25,036. The result of PS matching is depicted in Supplementary Figure S1.

The results of the logistic regression model for analyzing the preventive effect of BPs for osteoporotic fracture are summarized in Table 1. With this statistical model, we could not find a statistically significant preventive effect of BPs for osteoporotic fracture. However, with Cox proportional hazard regression analysis, BPs showed a statistically significant low hazard ratio (HR; Table 2). The preventive effect of BPs retained its statistical significance in various adjusted regression models. The use of BPs showed an HR of 0.890–0.895 compared with non-BP use (*p* < 0.001) in various regression models.

**Figure 1:**
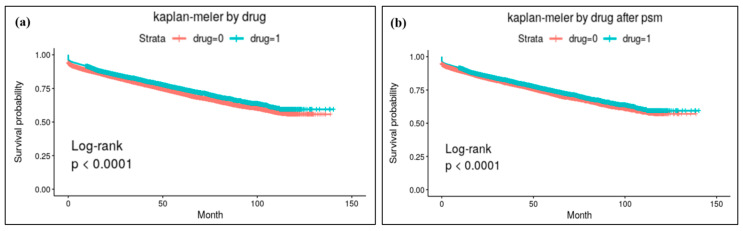
Results of Kaplan–Meier survival analysis for osteoporotic fracture: (**a**) analysis results with the original study population and (**b**) results with the population after PS matching. BPs showed statistically significant preventive effects for osteoporotic fracture in both models.

The results of log-rank analysis with Kaplan–Meier survival analysis are depicted in Figure 1. BPs showed statistically a significant preventive effect in the original population model and also in the model after PS matching.

**Figure 2:**
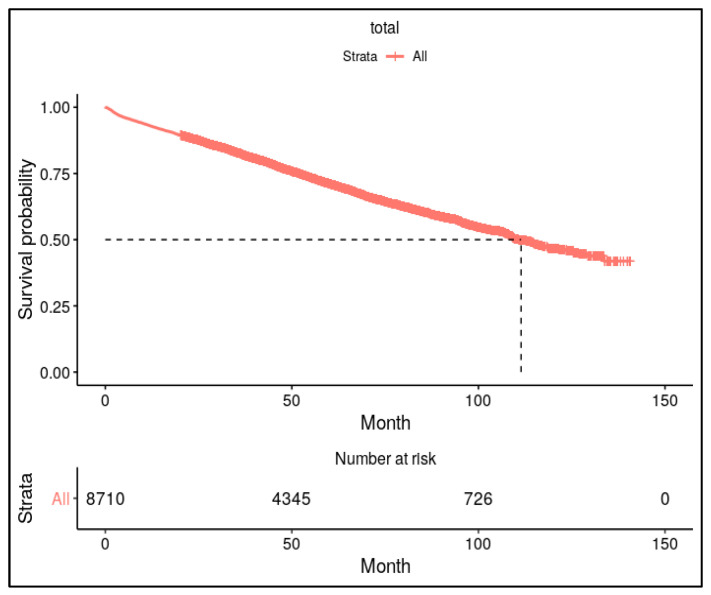
Kaplan–Meier survival analysis for mortality after diagnosing osteoporotic fracture in patients with AD. Approximately 50% of patients with osteoporotic fractures died within 10 years following the fracture.

We also performed additional analysis for mortality after the occurrence of osteoporotic fracture in patients with AD. The results of Kaplan–Meier survival analysis for mortality in AD patients having osteoporotic fracture are depicted in Figure 2.

The results of mortality analysis for each anatomical lesion of osteoporotic fracture are summarized in Table 3. Hip fracture showed a higher HR for death (HR = 2.036, 95% CI = 1.789–2.316, *p* < 0.001). Wrist fracture did not show a significant relation with mortality.

**Figure 3:**
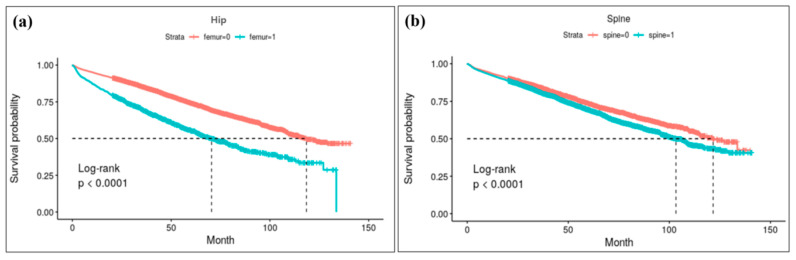
Result of Kapan–Meier survival analysis for each anatomical lesions of osteoporotic fracture: (**a**) survival curve for hip fracture. Log-rank analysis reveals a high risk of mortality for hip fracture patients; approximately 50% died within 6 years post-fracture, whereas for patients without fractures, it took approximately 11 years for 50% mortality. (**b**) survival curve following spine fracture.

Kaplan–Meier survival analysis for each anatomical lesion of osteoporotic fracture is depicted in Figure 3. Among AD patients with osteoporotic hip fractures, approximately 50% died within 6 years post-fracture, whereas in patients without fractures, it took approximately 11 years for 50% mortality to be observed over the same period (*p* < 0.001).

## 4. Discussion

The current study found that BPs showed significant preventive effects in preventing osteoporotic fractures, although the risk difference was not substantially high (use of BPs for osteoporotic fracture compared with the non-use group, HR of 0.890–0.895). More than 50% of patients with osteoporotic fracture died after fracture, and osteoporotic hip fracture showed greatest risk for death after fracture (HR = 2.036, 95% CI = 1.789–2.316, *p* < 0.001).

AD patients are particularly vulnerable to osteoporotic fractures due to their physical and cognitive impairments. Cognitive decline in AD can lead to poor judgment, impaired balance, and an increased risk of falls, which are primary contributors to fractures [14,15]. Additionally, reduced physical activity in AD patients exacerbates bone loss, further increasing their susceptibility to osteoporosis and fractures [6,11]. These combined factors give AD patients a significantly higher risk of osteoporotic fractures compared with the general older adult population [16].

The fracture-preventive effects of BPs have been well documented in prior studies. Black et al. demonstrated that annual administration of zoledronic acid significantly reduced vertebral and hip fractures in postmenopausal females with osteoporosis, emphasizing the protective role of BPs in high-risk populations [17]. Similarly, Cummings et al. showed that denosumab, a medication with a mechanism similar to that of BPs, effectively reduced fracture risks in postmenopausal females, suggesting the importance of antiresorptive therapy in managing osteoporosis [18]. Watts et al. reported that long-term BP use was associated with decreased fracture incidence, particularly in the hip and spine, among older adults [8]. Siris et al. also highlighted that accurate clinical diagnosis of osteoporosis plays a crucial role in identifying candidates for fracture prevention therapies, including BPs, highlighting the significance of timely intervention [19].

The results of the current study indicated that the effectiveness of BPs in preventing osteoporotic fractures was lower in patients with Alzheimer’s disease compared with the general older adult population. While previous studies have demonstrated an overall fracture prevention effect of approximately 21–38% in the general older adult population, the findings of this study showed a reduced preventive effect of approximately 11% in the AD patient group. This was presumed to be attributable to cognitive decline and activity limitations commonly observed in patients with AD.

Our study extended this understanding by demonstrating a statistically significant reduction in osteoporotic fractures among AD patients with the use of BPs. This aligns with findings from previous research but highlights a unique application of BP therapy in a cognitively impaired, physically restricted population. Compared with studies on the general population, our findings suggest that BP therapy could similarly benefit AD patients, despite their additional risk factors.

Research specific to AD patients remains sparse. However, recent studies have identified BP medications as potentially beneficial to neurodegenerative diseases such as Parkinson’s disease and AD [12,20]. These studies suggest that BP therapy may be broadly beneficial in neurocognitive disorders, even if direct evidence in AD is limited. Adami et al. further emphasized the importance of monitoring treatment effectiveness in osteoporosis patients to ensure sustained protection against fractures, particularly in vulnerable populations [21].

Osteoporotic fractures are known to significantly increase mortality rates in older adult populations. Haentjens et al. reported that hip fractures doubled the risk of mortality within one year compared with age-matched controls [22]. Sattui and Saag highlighted that vertebral fractures were associated with a 20% increased mortality risk within five years [23]. Additionally, Bliuc et al. demonstrated that fracture-related mortality rates remained elevated for up to a decade post-fracture [24]. The results of this study showed that 50% of AD patients who sustained an osteoporotic fracture died within 10 years, and 50% of those with hip fractures died within six years. Corroborating the current literature, these results make it evident that AD patients experience disproportionately higher mortality rates, likely due to their compounded physical and cognitive vulnerabilities.

Despite these findings, this study has several limitations. First, because this study utilized the claims data from the Korean national public database, it was impossible to ascertain actual medication adherence or compliance, and it could not use concepts of percentage of days covered, which may influence the outcomes. Second, the study relied on diagnostic codes for osteoporosis rather than precise bone mineral density (BMD) measurements, which limited the ability to stratify fracture risks based on bone health severity. Third, although propensity score matching was conducted to reduce confounding, the potential for residual confounding due to unmeasured variables cannot be entirely excluded. Additionally, Imaz et al. pointed out that adherence to therapy is a critical factor influencing outcomes, which could not be fully accounted for in our analysis [25].

In conclusion, our study demonstrated that continuous BP use was associated with reducing osteoporotic fractures in AD patients. Furthermore, AD patients who experience osteoporotic fractures have significantly higher mortality rates, especially hip fractures, emphasizing the importance of fracture prevention in this vulnerable group. These findings contribute valuable insights into osteoporosis management in neurodegenerative disorders, warranting further research into tailored interventions for AD populations.

## Figures and Tables

**Table 1:** Logistic regression model for analyzing the preventive effect of BPs for osteoporotic fracture.

	Unmatched Sample (n = 43,469)			Matched Sample (n = 25,036)		
	OR	95% CI	*p*-Value	OR	95% CI	*p*-Value
Unadjusted	0.956	0.910–1.004	0.070	0.989	0.933–1.049	0.708
Model 1	1.007	0.958–1.058	0.795	0.992	0.935–1.053	0.793
Model 2	1.007	0.958–1.058	0.787	0.992	0.935–1.053	0.802
Model 3	1.005	0.956–1.057	0.843	0.995	0.938–1.056	0.879

Model 1: sex + age + region + income level. Model 2: model 1 + myocardial infarction + CHF + peripheral vascular + cerebrovascular + dementia + chronic pulmonary + rheumatologic + peptic ulcer + mild liver + DM + hemiplegia + renal + lymphoma + severe liver + solid tumor + AIDS + hyperparathyroidism + thyrotoxicosis + IBD + ankylosing spondylitis + SLE + multiple myeloma. Model 3: model 2 + height + weight + waist + BP_SYS + BP_DIA + proteinuria + HbA1c + FBS + total cholesterol + triglyceride + HDL + LDL + AST_SGOT + ALT_SGPT + r-GTP + smoking + high alcohol intake + PAS + low BMI.

**Table 2:** Cox proportional hazard regression model for analyzing the preventive effect of BPs for osteoporotic fracture.

	Unmatched Sample (n = 43,469)			Matched Sample (n = 25,036)		
	HR	95% CI	*p*-Value	HR	95% CI	*p*-Value
Unadjusted	0.856	0.820–0.893	<0.001	0.894	0.850–0.942	<0.001
Model 1	0.906	0.868–0.946	<0.001	0.890	0.845–0.937	<0.001
Model 2	0.906	0.868–0.946	<0.001	0.890	0.846–0.937	<0.001
Model 3	0.906	0.868–0.946	<0.001	0.895	0.850–0.942	<0.001

Model 1: drug + sex + age + region + income level. Model 2: model 1 + myocardial infarction + CHF + peripheral vascular + cerebrovascular + dementia + chronic pulmonary + rheumatologic + peptic ulcer + mild liver + DM + hemiplegia + renal + lymphoma + severe liver + solid tumor + AIDS + hyperparathyroidism + thyrotoxicosis + IBD + ankylosing spondylitis + SLE + multiple myeloma. Model 3: model 2 + height + weight + Waist + BP_SYS + BP_DIA + proteinuria + HbA1c + FBS + total cholesterol + triglyceride + HDL + LDL + AST_SGOT + ALT_SGPT + r-GTP + smoking + high alcohol intake + PAS + low BMI.

**Table 3:** Cox proportional hazard regression model for mortality following osteoporotic fracture.

	Unadjusted Model			Adjusted Model		
	HR	95% CI	*p*-Value	HR	95% CI	*p*-Value
Hip	1.973	1.805–2.156	<0.001	2.036	1.789–2.316	<0.001
Spine	1.221	1.131–1.318	<0.001	1.465	1.305–1.644	<0.001
Wrist	0.551	0.483–0.629	<0.001	1.098	0.933–1.291	0.260

Adjusted model: hip + spine + wrist + sex + age + region + income level + myocardial infarction + CHF + peripheral vascular + cerebrovascular + dementia + chronic pulmonary + rheumatologic + peptic ulcer + mild liver + DM + hemiplegia + renal + lymphoma + severe liver + solid tumor + AIDS + hyperparathyroidism + thyrotoxicosis + IBD + ankylosing spondylitis + SLE + multiple myeloma + height + weight + waist + BP_SYS + BP_DIA + proteinuria + HbA1c + FBS + total cholesterol + triglyceride + HDL + LDL + AST_SGOT + ALT_SGPT + r-GTP + smoking + high alcohol intake + PAS + low BMI.

## Data Availability

The access period for scholarly use of the government national database used in this study has ended. Therefore, no data are available from after the current study analysis was finished.

## Supplementary Materials

The following supporting information can be downloaded at https://www.mdpi.com/article/10.3390/jcm14020300/s1: Table S1. Demographic characteristics of the patients. Figure S1. Results of propensity score matching.
