# Supplementary material for: Analysis of Preventive Effect of Bisphosphonate for Osteoporotic Fracture in Patients with Alzheimer’s Disease and Patient Mortality
Source: J Clin Med. 2025 Jan 7;14(2):300. doi: 10.3390/jcm14020300 (PMC11766000; doi:10.3390/jcm14020300)

Supplementary Table S1. Demographic Characteristics of the Patients

	Unmatched sample(n=43,469)				Matched sample(n=25,036)			
	Drug (n=12,518)	Non-drug (n=30,951)	p-value	SMD	Drug (n=12,518)	Non-drug (n=12,518)	p-value	SMD
Age (year)	71.5±7.9	73.2±8.0	<0.001	-0.209	71.5±7.9	71.5±8.1	0.845	-0.003
Sex (Male)	2376(19.0%)	8039(26.0%)	<0.001	0.070	2376(19.0%)	2283(18.2%)	0.135	-0.007
Region (Urban)*	9767(78.0%)	23402(75.6%)	<0.001	-0.024	9767(78.0%)	9779(78.1%)	0.867	0.001
Income**								
-1(lowest)	3159(25.2%)	7296(23.6%)		0.017	3159(25.2%)	3121(24.9%)		0.003
-2	1897(15.2%)	4796(15.5%)	0.004	-0.003	1897(15.2%)	1928(15.4%)	0.459	-0.003
-3	3007(24.0%)	7635(24.7%)		-0.007	3007(24.0%)	3097(24.7%)		-0.007
-4(highest)	4455(35.6%)	11224(36.3%)		-0.007	4455(35.6%)	4372(34.9%)		0.007
Comorbidity								
-Myocardial infarction	92(0.7%)	236(0.8%)	0.811	0.000	92(0.7%)	83(0.7%)	0.544	0.001
-CHF	321(2.6%)	909(2.9%)	0.037	-0.004	321(2.6%)	331(2.6%)	0.721	-0.001
-Peripheral vascular	627(5.0%)	1531(4.9%)	0.806	0.001	627(5.0%)	635(5.1%)	0.840	-0.001
-Cerebrovascular	944(7.5%)	2313(7.5%)	0.823	0.001	944(7.5%)	923(7.4%)	0.630	0.002
-Dementia	2956(23.6%)	7535(24.3%)	0.110	-0.007	2956(23.6%)	3026(24.2%)	0.306	-0.006
-Chronic pulmonary	427(3.4%)	1093(3.5%)	0.556	-0.001	427(3.4%)	431(3.4%)	0.917	0.000
-Rheumatologic	364(2.9%)	873(2.8%)	0.643	0.001	364(2.9%)	385(3.1%)	0.458	-0.002
-Peptic ulcer	400(3.2%)	912(2.9%)	0.180	0.003	400(3.2%)	387(3.1%)	0.664	0.001

-Mild liver	604(4.8%)	1525(4.9%)	0.673	-0.001	604(4.8%)	592(4.7%)	0.744	0.001
-DM	747(6.0%)	1709(5.5%)	0.072	0.005	747(6.0%)	721(5.8%)	0.501	0.002
-Hemiplegia	361(2.9%)	843(2.7%)	0.374	0.002	361(2.9%)	370(3.0%)	0.764	-0.001
-Renal	13(0.1%)	55(0.2%)	0.103	-0.001	13(0.1%)	8(0.1%)	0.383	0.000
-Lymphoma	200(1.6%)	502(1.6%)	0.889	0.000	200(1.6%)	197(1.6%)	0.919	0.000
-Severe liver	40(0.3%)	78(0.3%)	0.261	0.001	40(0.3%)	37(0.3%)	0.819	0.000
-Solid tumor	29(0.2%)	95(0.3%)	0.218	-0.001	29(0.2%)	30(0.2%)	1.000	0.000
-AIDS	1(0.0%)	2(0.0%)	0.999	0.000	1(0.0%)	1(0.0%)	1.000	0.000
-Hyperparathyroidism	37(0.3%)	69(0.2%)	0.199	0.001	37(0.3%)	37(0.3%)	0.720	0.000
-Thyrotoxicosis	163(1.3%)	408(0.2%)	0.931	0.000	163(1.3%)	161(1.3%)	0.955	0.000
-IBD	16(0.1%)	46(0.1%)	0.704	0.000	16(0.1%)	14(0.1%)	0.855	0.000
-Ankylosing spondylitis	38(0.3%)	81(0.3%)	0.512	0.000	38(0.3%)	39(0.3%)	1.000	0.000
- SLE	17(0.1%)	48(0.2%)	0.738	0.000	17(0.1%)	19(0.2%)	0.868	0.000
- Multiple myeloma	3(0.0%)	5(0.0%)	0.878	0.000	3(0.0%)	1(0.0%)	0.617	0.000
Hight	153.4±7.6	154.1±7.9	<0.001	-0.095	153.4±7.6	153.3±7.4	0.522	0.008
Weight	55.7±8.9	56.1±9.3	<0.001	-0.054	55.7±8.9	55.7±9.0	0.624	-0.006

Waist	81.4±8.7	81.7±8.9	<0.001	-0.042	81.4±8.7	81.5±8.8	0.170	-0.017
BP_SYS	127.7±16.2	128.2±16.3	0.003	-0.032	127.7±16.2	127.9±16.2	0.290	-0.013
BP_DIA	76.6±10.0	76.9±10.1	0.033	-0.023	76.6±10.0	76.7±10.1	0.430	-0.010
Proteinuria	1.1±0.5	1.1±0.5	0.001	-0.037	1.1±0.5	1.1±0.5	0.670	-0.005
HbA1c	12.9±1.3	12.9±1.4	<0.001	-0.039	12.9±1.3	12.9±1.4	0.806	-0.003
FBS	103.8±27.5	105.5±30.8	<0.001	-0.062	103.8±27.5	103.7±27.9	0.765	0.004
TOT_CHOL	193.1±43.2	193.6±44.3	0.308	-0.011	193.1±43.2	193.0±40.6	0.847	0.002
Triglyceride	131.6±75.4	134.2±78.8	0.001	-0.034	131.6±75.4	132.2±73.1	0.504	-0.008
HDL	54.5±20.4	54.0±20.6	0.027	0.024	54.5±20.4	54.4±22.6	0.703	0.005
LDL	112.8±41.4	113.7±49.1	0.065	-0.021	112.8±41.4	112.9±41.5	0.819	-0.003
AST_SGOT	25.8±14.5	26.0±15.0	0.228	-0.013	25.8±14.5	25.9±14.0	0.613	-0.006
ALT_SGPT	21.6±15.9	21.3±16.5	0.112	0.017	21.6±15.9	21.6±14.6	0.942	-0.001
r_GTP	28.5±39.9	30.0±43.4	0.001	-0.036	28.5±39.9	28.5±38.1	0.979	0.000
Smoking								
-non	10919(87.2%)	25952(83.8%)	<0.001	0.034	10919(87.2%)	10951(87.5%)	0.686	-0.003
-quit smoking	951(7.6%)	2983(9.6%)		-0.020	951(7.6%)	915(7.3%)		0.003
-smoking	648(5.2%)	2016(6.5%)		-0.013	648(5.2%)	652(5.2%)		0.000
High_alcohol_intake			0.005	-0.004			0.190	0.002
-non or mild drunken	12377(98.9%)	30493(98.5%)			12377(98.9%)	12399(99.0%)		
-heavy drunken	141(1.1%)	458(1.5%)			141(1.1%)	119(1.0%)		
PAS	2.5±3.3	2.4±3.2	0.003	0.031	2.5±3.3	2.5±3.3	0.475	0.009

low_BMI	577 (4.6%)	1693 (5.5%)	<0.001	-0.009	577 (4.6%)	583 (4.7%)	0.881	-0.001

Results were given with mean and standard deviation or number of patients and percentage

*Region was consist of Urban and Rural

**lowest level is 1 and highest level is 4.

Supplementary Figure S1. Result of propensity score matching

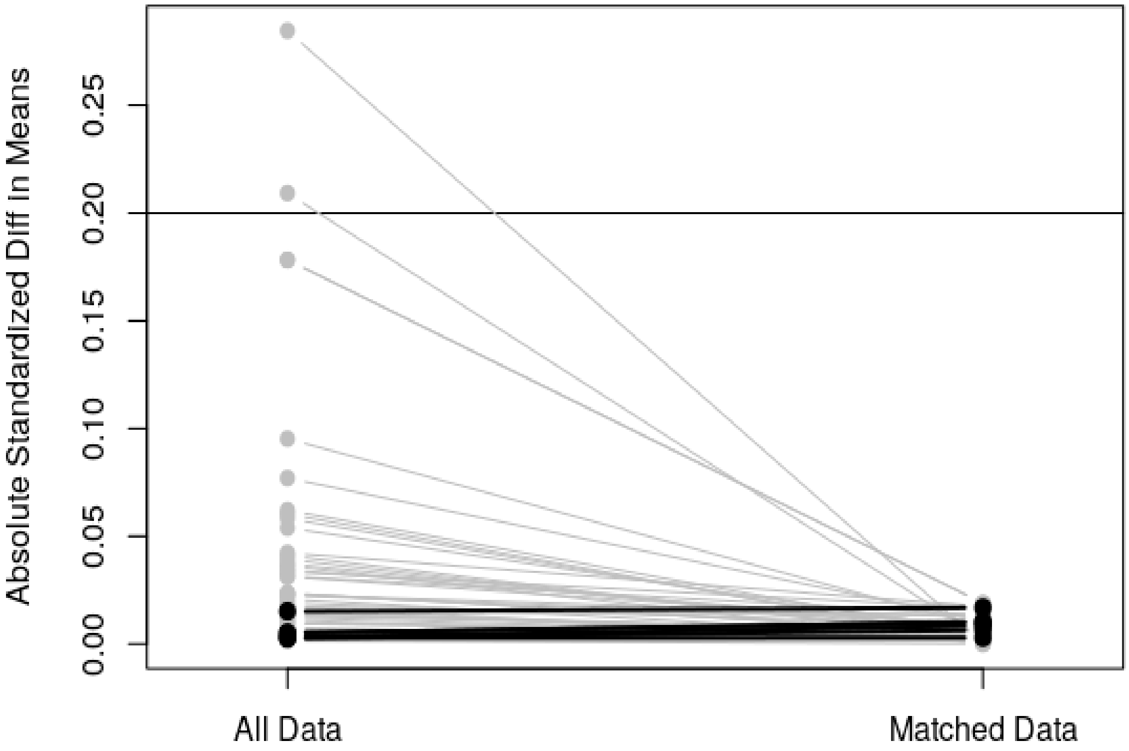

Supplement: Supplementary file 1 [file jcm-14-00300-s001.zip › jcm-3380461-supplementary.pdf]
